# Structure, Antimicrobial Activities and Mode of Interaction with Membranes of Bovel Phylloseptins from the Painted-Belly Leaf Frog, *Phyllomedusa sauvagii*

**DOI:** 10.1371/journal.pone.0070782

**Published:** 2013-08-13

**Authors:** Zahid Raja, Sonia André, Christophe Piesse, Denis Sereno, Pierre Nicolas, Thierry Foulon, Bruno Oury, Ali Ladram

**Affiliations:** 1 UPMC Univ Paris 06, ER3 Biogenèse des Signaux Peptidiques (BIOSIPE), Paris, France; 2 Institut de Recherche pour le Développement (IRD), Unité Mixte de Recherche IRD 224-CNRS 5290-Univ Montpellier 1 et 2, Maladies infectieuses et Vecteurs: écologie, génétique, évolution et contrôle (MiVegec), Montpellier, France; 3 UPMC Univ Paris 06, IFR 83 Plate-forme Ingénierie des Protéines et Synthèse Peptidique, Paris, France; University of Rouen, France, France

## Abstract

Transcriptomic and peptidomic analysis of skin secretions from the Painted-belly leaf frog *Phyllomedusa sauvagii* led to the identification of 5 novel phylloseptins (PLS-S2 to -S6) and also of phylloseptin-1 (PSN-1, here renamed PLS-S1), the only member of this family previously isolated in this frog. Synthesis and characterization of these phylloseptins revealed differences in their antimicrobial activities. PLS-S1, -S2, and -S4 (79–95% amino acid sequence identity; net charge  = +2) were highly potent and cidal against Gram-positive bacteria, including multidrug resistant *S. aureus* strains, and killed the promastigote stage of *Leishmania infantum*, *L. braziliensis* and *L. major*. By contrast, PLS-S3 (95% amino acid identity with PLS-S2; net charge  = +1) and -S5 (net charge  = +2) were found to be almost inactive against bacteria and protozoa. PLS-S6 was not studied as this peptide was closely related to PLS-S1. Differential scanning calorimetry on anionic and zwitterionic multilamellar vesicles combined with circular dichroism spectroscopy and membrane permeabilization assays on bacterial cells indicated that PLS-S1, -S2, and -S4 are structured in an amphipathic α-helix that disrupts the acyl chain packing of anionic lipid bilayers. As a result, regions of two coexisting phases could be formed, one phase rich in peptide and the other lipid-rich. After reaching a threshold peptide concentration, the disruption of lipid packing within the bilayer may lead to local cracks and disintegration of the microbial membrane. Differences in the net charge, α-helical folding propensity, and/or degree of amphipathicity between PLS-S1, -S2 and -S4, and between PLS-S3 and -S5 appear to be responsible for their marked differences in their antimicrobial activities. In addition to the detailed characterization of novel phylloseptins from *P. sauvagii*, our study provides additional data on the previously isolated PLS-S1 and on the mechanism of action of phylloseptins.

## Introduction

The dermaseptin superfamily contains several families of host defense peptides that are synthetized in the skin of Hylidae frogs [1], [2], [3]. The peptides are small, 10 to 50 amino acid residues long, cationic, and act in different ways, although disrupting and permeabilizing the anionic bacterial cell membrane is the most frequent [4]–[7]. These peptide families include the dermaseptins (*stricto sensu*), the plasticins, the dermatoxins, the phylloxins, the phylloseptins, the raniseptins, the caerins, the caerin-related peptides, the hyposins, the fallaxidins, the frenatins, and the aureins. It has been speculated that the diversification of antimicrobial peptides within a species is part of an evolutionary strategy for providing frogs with the maximum protection against a wide range of microorganisms [3], [8], [9]. Thus, the impressive interspecies variations in the expression of skin antimicrobial peptides may be exploited for discovering new antimicrobial molecules targeting specific microorganisms for which the therapeutic armamentarium is scarce. In addition, the discovery of new peptides with novel structural and biochemical properties may also shed light on the exact roles of various parameters, such as net charge, percent of α-helical/β-sheet structure, and amphipathicity, on the ability of antimicrobial peptides to bind to and disrupt microbial membranes. Moreover, the strategy that the frogs have evolved over millions of years for the generation and design of an enormous array of small peptide antibiotics, each with high potency and specificity for particular microorganisms, may be adaptable for use *in vitro*.

The 16–19 amino acid residue phylloseptins (PLSs) are a new emerging family of cationic antimicrobial peptides that belong to the dermaseptin superfamily, and whose members have been isolated from various genera of the Phyllomedusinae subfamily (*Agalychnis, Phasmahyla*, and *Phyllomedusa*) [2], [10]–[16]. Like the archetypal dermaseptins (*sensu stricto*), these peptides seem to occur in multiple paralogous forms in each species with small differences in amino acid composition. Although only a few of these peptides were properly tested against various microorganisms, significant differences in antimicrobial activities and target microorganism specificities have been observed between orthologous peptides, as well as paralogous peptides. For instance, PLS-H1 from *Phyllomedusa hypochondrialis* and phylloseptin-1 (PSN-1) from *Phyllomedusa sauvagii* (63% amino acid sequence identity) exhibited marked differences in their potencies to inhibit the growth of *Escherichia coli* (minimal inhibitory concentration, MIC  = 8 µM and 80 µM, respectively) [10], [15], whereas PLS-H1 -H2, and -H3 (74% sequence identity) demonstrated almost similar activities against Gram-negative and Gram-positive bacteria (MIC ranging from 2 to 8 µM) [10], [17]. PLS-L1 from *Hylomantis lemur* is highly potent against the Gram-positive bacteria *Staphylococcus aureus* (MIC  = 8 µM) [13], whereas PLS-L2 is only weakly active (MIC  = 50 µM) but exhibit insulin-releasing activity [14]. Interestingly, some phylloseptins (PLS-H1; PLS-O1 and -O2 from *Phyllomedusa oreades*) demonstrated *in vitro* activity against the protozoan parasites *Leishmania amazonensis* promastigotes, *Plasmodium falciparum* (rings, trophozoites, and schizonts), and *Trypanosoma cruzi* (trypomastigotes) [10], [18], making phylloseptins potential candidates for the treatment of malaria, leishmaniasis, and Chagas disease. The phylloseptins are Lys/His-rich antimicrobial peptides, most of them carrying 1 to 3 His residues that may lead to a variation of the effective net charge and biological activity at physiological and acidic pHs, and can be predicted to adopt an amphipathic α-helical structure in membrane-mimetic environment [17]. Although the mechanisms by which phylloseptins act against microbial cells are unknown, it is speculated that the cationic peptides first bind to the anionic microbial membrane surface and then adopt a helical amphipathic structure that promotes their insertion into the membrane, causing the collapse of the membrane at a critical peptide concentration.

In the present study, we have used the conservation of the preproregion sequences of the preprodermaseptin transcripts to identify and isolate five new members of the phylloseptin family (PLS-S2 to -S6) in the South American hylid frog *Phyllomedusa sauvagii* (subfamily: Phyllomedusinae), in addition to the previously isolated phylloseptin-1 (PSN-1, here renamed PLS-S1) [15] (Table 1). A detailed characterization of these synthetic phylloseptins was undertaken, except for PLS-S6, a paralog with physicochemical properties identical to PLS-S1. Structure and activities were determined using circular dichroism and antimicrobial assays against various microorganisms, including Gram-positive and Gram-negative bacteria, yeasts, and *Leishmania* parasites. To address the mechanism of action, we have used bacterial time-kill and permeabilization assays, and differential scanning calorimetry on multilamellar vesicles composed of anionic and zwitterionic lipids as models for prokaryotic and eukaryotic plasma membranes.

**Table 1:** Primary structure of phylloseptins-S identified in the skin of *P. sauvagii*.

Phyllo septin	Sequence	Net charge	<H>	<µH>	Fraction
PLS-S1	F**L**SL**IP**HIV**S**GVASIAKHF_a_	+2	0.81	0.55	54–56
PLS-S2	F**L**SL**IP**HIV**S**GVASLAKHF_a_	+2	0.80	0.55	54–56
PLS-S3	F**L**SL**IP**HIV**S**GVASLAIHF_a_	+1	0.95	0.43	ND
PLS-S4	F**L**SM**IP**HIV**S**GVAALAKHL_a_	+2	0.79	0.52	53
PLS-S5	L**L**GM**IP**VAI**S**AISALSKL_a_	+2	0.85	0.35	62
PLS-S6	F**L**SL**IP**HIV**S**GVASIAKHL_a_	+2	0.80	0.55	ND

Identical amino acid residues are indicated in bold. a: amide; <H>: Hydrophobicity; <µH>: Hydrophobic moment. The net charge (pH 7.4) and the original HPLC fraction are indicated. ND: not detected. The C-terminal amidation of PLS-S3 and -S5 is based on the presence of the Gly residue (amide donor) at the C-terminus of the PLS precursor and on the fact that all members of the PLS family are amidated.

## Materials and Methods

### Frog species

Specimens of *P. sauvagii* were obtained from a commercial source (La Ferme Tropicale, Paris, France). Frogs were bred as previously described [19]. They were housed in large wooden cages (120×90×90 cm), covered on three sides by plastic mosquito netting. Phyllodendron, Potos, and Dracena were used as perches, and water bowls were provided for nocturnal bathing. The frogs were fed crickets. The protocol of animal handling and treatment was performed in accordance with the guidelines of the animal ethics committee of the French Ministry of Agriculture, Veterinary Department for Animal Health and Protection, under the supervision of authorized investigators (agreement N I-75UPMC-F1-07). Our study does not involve *in vivo* experiments and animal suffering.

### Cloning of *P. sauvagii* phylloseptin cDNA precursors

Skin secretions of one adult specimen of *P. sauvagii* were collected by gentle squeezing of the latero-dorsal portion of the skin of the living frog, as previously described [19] and dilution in 1 ml sterile diethylpyrocarbonate (DEPC)-H_2_O. The solution was rapidly frozen in liquid nitrogen and lyophilized. Poly(A)^+^ RNA was isolated from the dry material (5 mg) using the Micro-FastTrack mRNA isolation kit (Invitrogen) and transcribed into cDNAs with an oligo(dT) primer (Advantage RT-for-PCR kit, Clontech) according to the protocol of the manufacturers. PCR was performed using a set of specific primers (5′-TGACCTTCAGTACCCAGCACTTTC-3′/5′-GTGGTACATAATTGATAATTGTGCT-3′) matching the conserved 5′- and 3′-UTR of preprodermaseptins, respectively. The following cycling procedure was used: initial denaturation, 2 min at 94°C; 30 cycles of 45 s at 94°C, 1 min at 49°C, 3 min at 72°C; final extension, 10 min at 72°C. PCR products were gel purified (NucleoSpin Extract, Macherey-Nagel), cloned into the pGEM-T easy vector system (Promega) and sequenced (Beckman Coulter Genomics SA). The nucleotide sequences of pre-prophylloseptins (ppPLSs) described in this study have been deposited in the EMBL Nucleotide Sequence Database under the accession numbers **AM903077** (ppPLS-S1), **AM903078** (ppPLS-S2), **AM903079** (ppPLS-S3), **AM903080** (ppPLS-S4), **AM903081** (ppPLS-S5), **HE974361** (ppPLS-S6).

### Identification of mature phylloseptins in the skin extract of *P. sauvagii*

80 mg of a frozen acidic lyophilized extract of *P. sauvagii* skin secretions were obtained according to a previous described protocol [20] and were prepurified on a Sep-Pak C-18 cartridge eluted with 70% acetonitrile (ACN) in 0.1% trifluoroacetic acid (TFA)/H_2_O. The prepurified extract (7 mg) was lyophilized, reconstituted in 0.1% TFA/H_2_O and fractionated by reversed-phase high performance liquid chromatography (RP-HPLC) on a semi-preparative Nucleosil C-18 column (5 µm, 250×10 mm, Interchim) eluted at 4 mL/min with a 0–70% linear gradient of acetonitrile in 0.1% TFA/water (1% ACN/min). Fractions corresponding to 4 mL were collected and analyzed by MALDI-TOF-MS (Voyager DE-PRO Applied Biosystems) using the matrix α-cyano-4-hydroxycinnamic acid. The MS positive ion spectra were carried out in the reflector mode with external calibration using the 4700 Standard kit. The primary structure of mature PLSs was confirmed by MALDI-TOF-TOF (4700 Proteomic Analyzer, Applied Biosystems). The protonated molecule of the precursor was selected in the window (−5u,+5u) and fragmented using N_2_ as the collision gas (2×10^−7^ Torr, collision energy 1 keV).

### Solid phase peptide synthesis

Carboxamidated phylloseptins (PLS-S1, -S2, -S3, -S4 and -S5) were synthesized using solid-phase FastMoc chemistry procedure on an Applied Biosystems 433A automated peptide synthesizer as described [20]. Resin and Fmoc-protected amino acids were purchased from Iris Biotech GMBH, and solvents from Carlo Erba. Briefly, carboxamidated peptides were prepared on a 4-methylbenzhydrylamine polystyrene resin (Rink Amide MBHA PS resin) substituted at 0.81 mmol/g. Activation (30 min) of Fmoc amino acids (10 molar excess) was realized with 2-(1H-b enzotriazol-1-yl)-1,1,3,3-tetramethyluronium hexafluorophosphate (0.5 M HBTU solubilized in dimethylformamide) and diisopropylethylamine (2 M) in N-methylpyrrolidone (NMP). N-Fmoc protecting groups were removed with 20% piperidine in NMP. A systematic double-coupling protocol was used followed by a capping step with acetic anhydride performed at the end of each cycle. The peptidyl resin was cleaved and deprotected by incubation with an acidic cocktail (95% TFA, 2.5% triisopropylsilane, 2.5% water) for 3 h at room temperature (for PLS-S4 and -S5 containing a Met residue: 94% TFA, 1% triisopropylsilane, 2.5% ethanedithiol, 2.5% water). The resin was removed by filtering the resulting mixture, and the crude peptides were precipitated with methyl-tert-butyl ether (MTBE) at 4°C. They were recovered by centrifugation (3,000×g, 15 min, 4°C), washed 3 times with cold MTBE, dried under a stream of nitrogen, dissolved in 10% acetic acid, and lyophilized. The lyophilized crude peptides were purified by RP-HPLC on a Phenomenex Luna® C18(2) semi-preparative column (10 µm, 250×10 mm) eluted at a flow rate of 5 mL/min by a 20–70% linear gradient of ACN (0.07% TFA) in 0.1% TFA/water (1% ACN/min). The homogeneity and identity of the synthetic peptide were assessed by matrix-assisted laser desorption/ionization-time of flight (MALDI-TOF) mass spectrometry (Voyager DE-PRO Applied Biosystems) and RP-HPLC on a C18 analytical column (modulocart QS uptisphere 5 ODB, 5 mm, 250×4.6 mm, Interchim) using the above conditions with a flow rate of 0.75 mL/min.

### Circular dichroism spectroscopy

The far-ultraviolet circular dichroism (CD) spectra were recorded as described [20]. Phylloseptins-S (30 µM) were solubilized either in 80 mM sodium dodecyl sulfate (SDS) or in phosphate-buffered saline (PBS: 10 mM phosphate, pH 7.3) containing 1 mg/mL of large unilamellar vesicles (LUVs) of 1,2-dimyristoyl-sn-glycero-3-phosphocholine (DMPC) and 1,2-dimyristoyl-sn-glycero-3-phosphoglycerol (DMPG) (3∶1 mol/mol). DMPC and DMPG were purchased from Avanti Polar Lipids, Inc. Each spectrum was the average of four scans. CD measurements are reported as Δ/n, where Δ is the dichroic increment and n is the number of residues in the peptide.

### Antimicrobial activities

The effect of phylloseptins-S was evaluated against several bacterial strains and yeasts (Table 2). Bacteria were cultured in Luria-Bertani broth (LB), whereas yeasts were cultured in Yeast Peptone Dextrose medium (YPD), and minimal inhibitory concentrations (MICs) were determined by measuring the absorbance at 630 nm in 96-well microtitration plates, as described previously [21]. Leishmanicidal activity of phylloseptins-S was also examined against promastigotes of different *Leishmania* species: *Leishmania infantum* (strain MHOM/MA/67/ITMAP-263), *L. major* (MHOM/SU/73/5-ASKH) and *L. braziliensis* (MHOM/BR/75/M2904). Culture of promastigotes and determination of the growth inhibition were performed as previously described [20], [22], [23].

**Table 2:** Antimicrobial activity of phylloseptins-S.

	PLS-S1	PLS-S2	PLS-S3	PLS-S4	PLS-S5
			MIC (µM)		
**Gram-positive bacteria**					
*S. aureus* ATCC 25923	6.25	6.25	>200	6.25	25
*S. aureus* ST1065	6.25	6.25	>200	6.25	>100
*S. aureus* ATCC 43300^a^	6.25	6.25	>200	6.25	>100
*S. aureus* ATCC BAA-44^b^	6.25	6.25	>200	6.25	>100
*E. faecalis* ATCC 29212	25	25	>200	50	100
*S. pyogenes* ATCC 19615	3.12	1.56	12.5	3.12	ND
**Gram-negative bacteria**					
*E. coli* ATCC 25922	70	25	>200	25	>100
*E. coli* ML-35p	>100	30	>200	25	>100
*P. aeruginosa* ATCC 27853	>100	200	>200	100	>100
*A. baumannii* ATCC 19606	6.25	6.25	>200	6.25	ND
*K. pneumoniae* ATCC 13883	>100	25	>200	25	ND
**Yeasts**					
*C. parapsilosis* ATCC 22019	100	50	>200	50	>100
*S. cerevisiae*	12.5	6.25	>200	12.5	ND

			IC_50_ (μM)		
*Leishmania promastigotes*					
*L. infantum*	16.5	18.5	NA	22.0	NA
*L. major*	12.6	13.3	NA	18.0	NA
*L. braziliensis*	15.3	15.0	NA	17.2	NA

a Resistant to methicillin and oxacillin. ^b^Resistant to amoxicillin/clavulanic acid, cephalothin, ciprofloxacin, erythromycin, gentamicin, imipenem, oxacillin, penicillin, tetracycline, ampicillin, doxycycline, methicillin, azithromycin, ceftriaxone, clindamycin, lincomycin, perfloxacin, rifampin, and tobramycin. ND: not determined. NA: not active as it was not possible to reach IC_50_ at the highest concentration tested (60 µM). MICs and IC_50_ are expressed as average values from three independent experiments performed in triplicates. IC_50_ values were determined from a dose-response inhibition fit using GraphPad Prism 5.0.

### Cytotoxic activities

Hemolytic assays were performed using fresh human erythrocytes from a healthy donor according to a previously described protocol [20]. Cytotoxic activity of PLS-S was also determined on the human leukemia monocyte cell line THP-1. 80 µL of 6.25×10^5^ cells/mL were incubated for 72 h with 20 µL of peptide (3 to 50 µM, final concentrations) at 37°C and 5% CO_2_ in RPMI medium supplemented with 10% FCS. The cell viability was determined by adding 10 µL of methylthiazolyldiphenyl-tetrazolium bromide (MTT, 10 mg/mL) to the plate. After 4h incubation at 37°C, formazan was solubilized with 100 µL of a solution containing 50% isopropanol and 10% SDS, and the absorbance was measured at 570 nm. The lytic concentration 50 (LC_50_), which correspond to the peptide concentration producing 50% lysis, was determined with the GraphPad Prism® 5.0 software. Results were expressed as the mean of three independent experiments performed in triplicates.

### Time-kill assays

The time-kill kinetics of phylloseptins-S against the Gram-negative bacteria *E. coli* ML-35p and the Gram-positive strain *S. aureus* ST1065 were evaluated as previously described [20] with a dose of peptide corresponding to the MIC and two-fold above the MIC. Two experiments were realized in triplicates.

### Permeabilization of the cytoplasmic membrane of Gram-positive (*S. aureus* ST1065) and Gram-negative (*E. coli* ML-35p) bacteria

PLS-induced permeabilization of the cytoplasmic plasma membranes of *E. coli* ML-35p and *S. aureus* ST1065 was measured by the rate of production of *o*-nitrophenol (ONP) at 405 nm, following hydrolysis of o-nitrophenyl β-D-galactopyranoside (ONPG) by β-galactosidase [24]. Dermaseptin B2 (10 µM) [6] and PBS were used as positive and negative controls, respectively.

### Study of the interactions of phylloseptins-S with vesicular model membranes by differential scanning calorimetry

Differential scanning calorimetry (DSC) experiments were performed using DMPC and DMPG (Avanti Polar Lipids, Inc.) multilamellar vesicles (MLVs) according to the procedure previously described [25]. Twenty scans were run for each sample, with 10 min equilibration time between each scan. Thermodynamic values (Tm and ΔH) were estimated with the CpCalc software.

### Molecular dynamic simulations

The docking studies of PLS-S2 with *E. coli* membrane model were carried out using Hex 6.3 protein docking software with a scoring function based on shape complementarities and electrostatic potentials. File for *E. coli* cytoplasmic membrane [26] was accessed from Department of Chemical and Biomolecular Engineering, Maryland, website (http://terpconnect.umd.edu/~jbklauda/research/download.html). Structural files for linear and α-helical peptides were built with the Avogadro software 1.1.0. The structures were minimized using the conjugate gradient method and then were used for docking. For the non-structured peptide, a molecular dynamic simulation during 1 ns was performed on Abalone 1.8.53 using a flexible simple point-charge (SPC) water model [27]. The 3D model structures were visualized using Rasmol 2.7.5.2 software.

## Results

### cDNA cloning of phylloseptins-S from *P. sauvagii*

**Figure 1:**
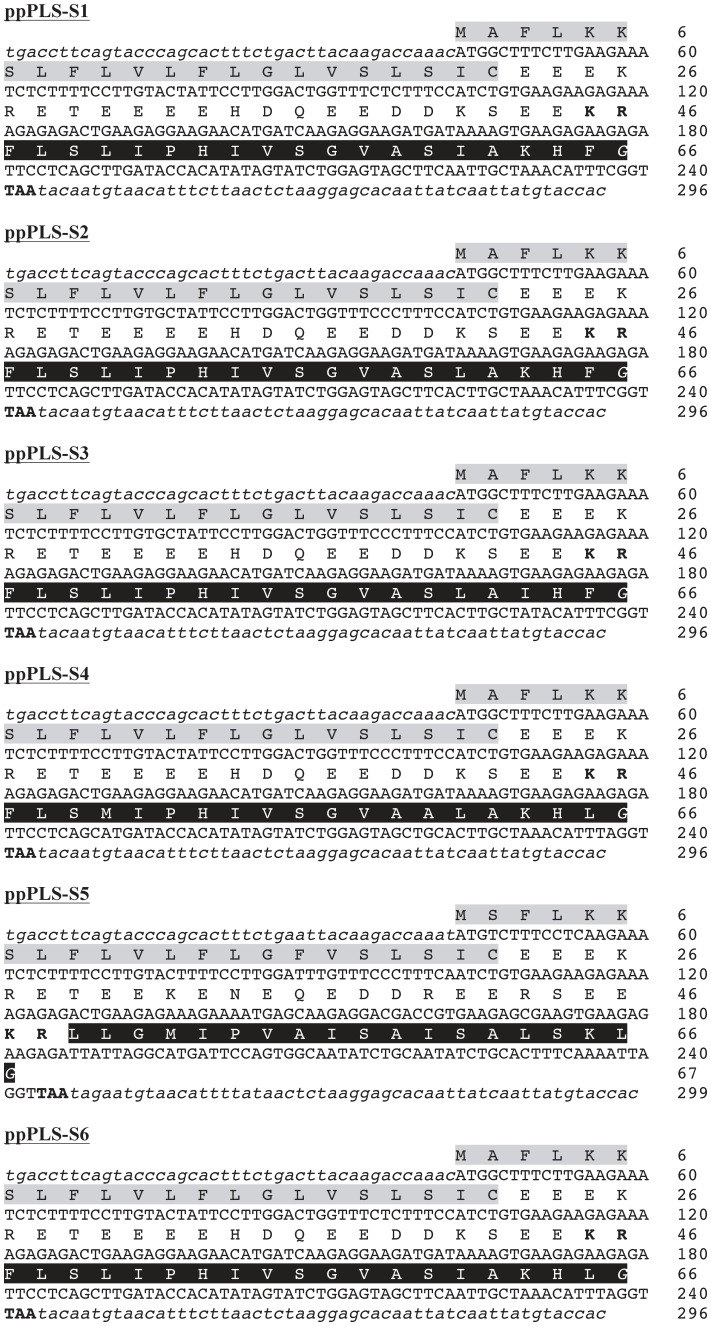
Complete nucleotide and deduced amino acid sequences of the cDNAs encoding *P. sauvagii* preprophylloseptin-S1 (ppPLS-S1, EMBL accession number: AM903077), -S2 (ppPLS-S2, AM903078), -S3 (ppPLS-S3, AM903079), -S4 (ppPLS-S4, AM903080), -S5 (ppPLS-S5, AM903081), and -S6 (ppPLS-S6, HE974361). The open reading frames (capital letters) contain the signal peptide (gray), followed by an acidic sequence ending with a pair of basic residues (in bold), and the phylloseptin progenitor sequence (black). The G residue (in italic) at the C-terminal of the progenitor sequence serves as an amide donor. The stop codon is indicated in bold. The partial 5′- and 3′- UTR are represented in italics and lowercase.

RT-PCR experiments were performed using *Phyllomedusa sauvagii* skin exudate mRNA as template and specific oligonucleotides designed to the conserved region (5′- and 3′-UTR) of previously cloned precursors of the dermaseptin superfamily. A 300-bp amplified fragment was obtained and revealed after cloning and sequencing six different full-ORF phylloseptin precursor cDNAs encoding 66–67 residue sequences starting with a Met codon and ending with a stop codon (Fig. 1). All the deduced amino acid sequences contain a 22-residue signal peptide followed by a 24–26-residue acidic propiece with a pair of basic residues (Lys-Arg) at its C-terminus. A single copy of the mature PLS progenitor sequence is found at the extreme C-terminus of the precursors flanking the acidic propiece. One of these cDNAs encodes phylloseptin-1 (PSN-1) [15] that we have renamed PLS-S1 (Table 1) in accordance with the established nomenclature [28]. The remaining five cDNAs correspond to novel phylloseptins-S and were designated as PLS-S2, -S3, -S4, -S5 and -S6 (Table 1). All these PLS-S precursors had the characteristic signature of the preproforms of the dermaseptin superfamily, i.e. a signal peptide ending with a Cys residue, an acidic propiece with a typical prohormone processing signal Lys-Arg at the C-terminus, and a single downstream copy of the mature peptide progenitor sequence. The PLS-S precursors end with a Gly residue that serves as an amide donor for the C-terminal residue of the mature PLS (see below).

### Purification and identification of phylloseptins-S in the skin of *P. sauvagii*

**Figure 2:**
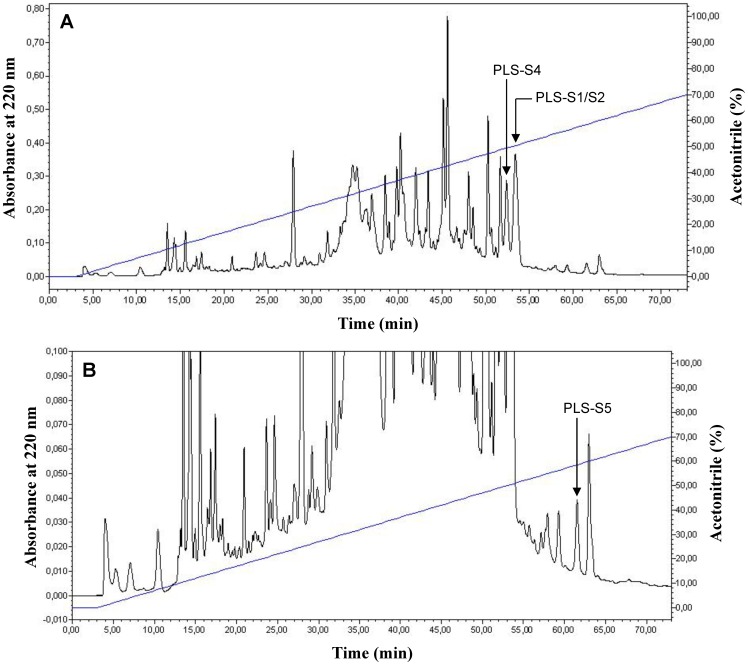
Reversed-phase HPLC chromatogram of *P. sauvagii* skin extract prepurified on a sep-pak C18 cartridge (A, full scale; B, zoom). The sample was injected on a semi-preparative Nucleosil C18 column eluted at 4 mL/min with a 0–70% linear gradient of acetonitrile in 0.1% TFA/water (1% ACN/min). Fractions of 4 mL were collected, lyophilized, and analyzed. The position of the mature PLSs is indicated by an arrow (PLS-S3 and PLS-S6 were not detected). The identification of PLSs was achieved by MALDI-TOF-MS and MS/MS (Supporting information, Fig. S1 A–C and S2 A–C).

Extracts from the skin of *P. sauvagii* were analyzed by reversed-phase HPLC to assess the presence of the predicted mature PLSs and the status of their C terminus *in vivo* (Fig. 2). Peptides with molecular masses matching those of PLS-S1, -S2, -S4, and -S5 were found by MALDI-TOF mass spectrometry (MALDI-TOF MS) (Fig. S1 A–C). The primary structure of the corresponding peptides (Table 1) was established by MALDI-TOF-TOF MS/MS from collision-induced dissociation of the parent ion and confirmed by the presence of the two-fragmentation series b and y and their amino acid sequences (Fig. S2 A–C). For each PLS-S, the sequence was obtained with remaining leucine/isoleucine indeterminations. PLS-S3 and -S6 were not detected probably due to their non-desorption or their presence in very small amount.

### Antimicrobial and cytotoxic activities of phylloseptins-S

**Figure 3:**
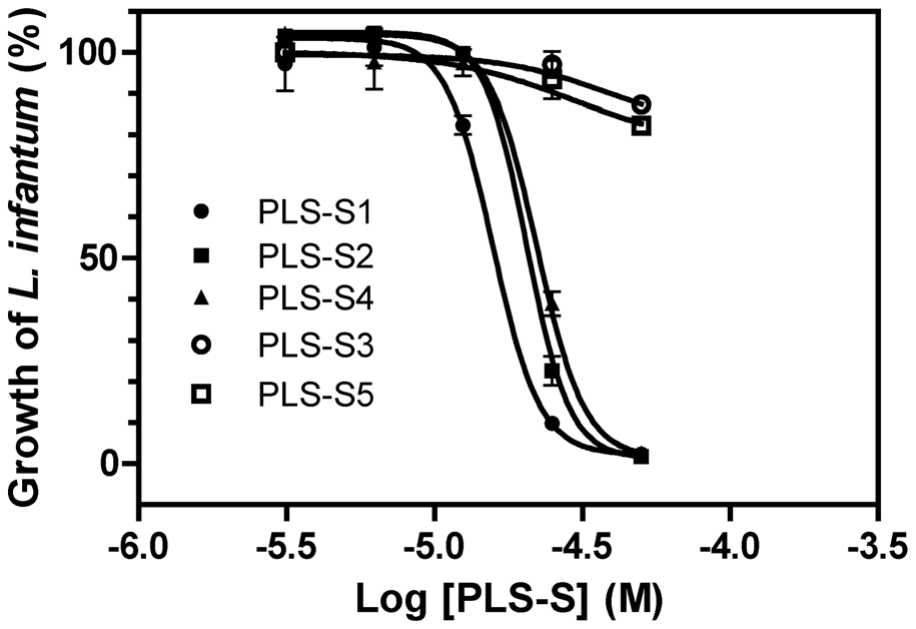
Activity of phylloseptins-S against *Leishmania infantum* promastigotes. IC_50_ values were determined from a dose-response inhibition fit using GraphPad Prism 5.0 (Table 2). Results represent the mean ± S.E.M. and are representative of three experiments carried out in triplicates.

The antimicrobial activity of synthetic PLS-S1 to -S5 was assayed in LB medium (pH 7) against several Gram-negative and Gram-positive bacteria, and multi-drug resistant *S. aureus* strains. As PLS-S6 is an analog of PLS-S1 (Leu instead of Phe at the C-terminus) with identical physicochemical properties (Table 1) and was not detected in HPLC fractions, the activity of this peptide was not evaluated. As indicated in Table 2, despite their paralogous relationships, the spectra of action of PLSs differ considerably. PLS-S1, -S2, and -S4 were highly active against Gram-positive bacteria with MIC in the range 1.56–6.25 μM, except *E. faecalis* for which a moderate (PLS-S1 and -S2, MIC  = 25 µM) or low (PLS-S4, MIC  = 50 µM) activity was observed. However, PLS-S1, -S2, and -S4 were less potent or devoid of activity against Gram-negative bacteria (MIC  = 25–30 µM) and yeasts (MIC  = 50–100 µM), except *A. baumannii* and *S. cerevisiae* for which a potent activity (MIC  = 6.25–12.5 µM) was observed. The dose-response profiles showed sharp curves in which inhibition of 0–100% was generated within a 1−2-fold peptide dilution (not shown). No visible colony was observed when MIC well contents (*S. aureus* ATCC 25923 and *E. coli* ATCC 25922) were spread on agar plates (37°C, overnight), indicating that the peptides were bactericidal. The antiparasitic activity of PLSs was also determined against three *Leishmania* species (*L. infantum, L. major, and L. braziliensis*). As indicated in Table 2 and Fig. 3, PLS-S1, -S2 and -S4 were highly potent against *Leishmania* species (IC_50_  = 13–22 μM). PLS-1, -S2 and -S4 have moderate hemolytic activities with a LC_50_ (25–40 µM) above the MIC values determined for most of the sensitive Gram-positive bacterial strains tested, but were toxic for human monocytes THP-1 (LC_50_ = 23 μM) at leishmanicidal concentrations (12–22 μM) (Table 3). PLS-S3 and -S5 were inactive against most of the bacteria and protozoa tested, except *S. pyogenes* (PLS-S3, MIC  = 12.5 µM) and *S. aureus* ATCC 25923 (PLS-S5, MIC  = 25 µM) (Table 2). Because PLS-S3 contains histidines as basic residues and that approximately only 11% of these residues are positively charged at pH 7, we have evaluated the antibacterial activity at pH 5 at which His residues are predominantly positively charged (∼91%). Interestingly, PLS-S3 was found to be active (MIC  = 50 µM) against *E. coli* ATCC 25922 (>200 µM at pH 7). Moreover, PLS-S1 another peptide containing His residues that is virtually inactive (MIC  = 70 µM) against *E. coli* ATCC 25922 was shown to be potent (MIC  = 12.5 µM) at acidic pH. These results indicate that pH influences the positive net charge of histidines with an impact on the antimicrobial activity.

**Table 3:** Cytotoxic activity of phylloseptins-S.

	PLS-S1	PLS-S2	PLS-S4
Human erythrocytes (LC_50_, µM)	39	25	33
Human THP-1 monocytes (IC_50_, µM)	23	22.5	23

LC_50_ and IC_50_ are expressed as average values from three independent experiments performed in triplicates. IC_50_ values were determined from a dose-response inhibition fit using GraphPad Prism 5.0.

### Killing kinetics of *E. coli* ML-35p and *S. aureus* ST1065

**Figure 4:**
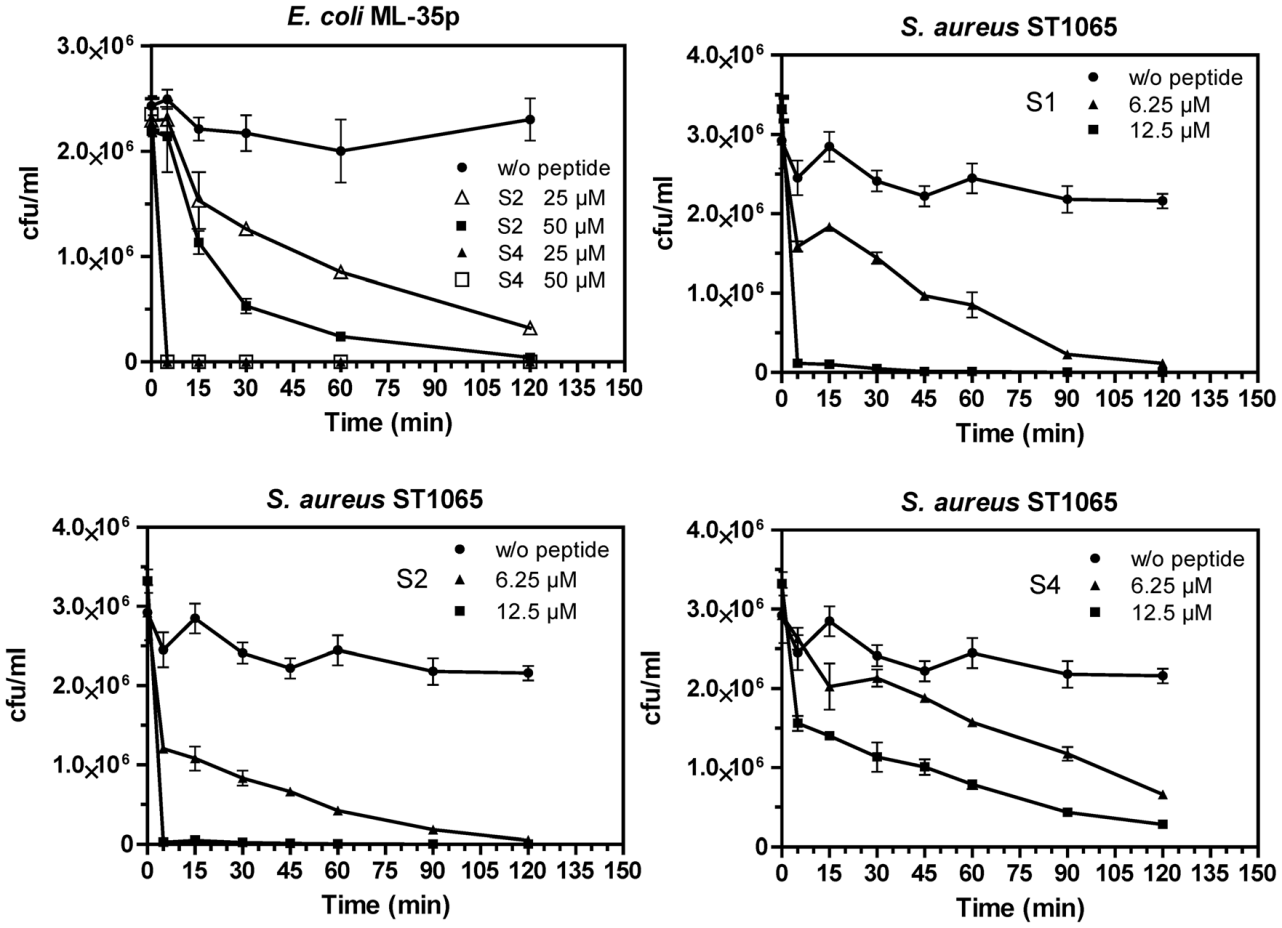
Time-kill curves of PLS-S1 (S1), -S2 (S2), and -S4 (S4). Bacteria (*E. coli* ML-35p and *S. aureus* ST1065, ∼2–3 10^6^ cfu/mL) were diluted in PBS and incubated with synthetic PLSs at concentrations corresponding to the MIC (*E. coli* ML-35p: 25 µM, *S. aureus* ST1065: 6.25 µM) and two-fold above the MIC. Controls correspond to bacteria incubated in PBS without peptide. The data are the means ± S.E.M. of two experiments carried out in triplicates.

The killing effect of PLSs toward *S. aureus* ST1065 (PLS-S1, -S2 and -S4) and *E. coli* ML-35p (PLS-S2 and -S4) was investigated at concentrations corresponding to the MIC (6.25 µM and 25 µM, respectively) and approximately two-fold above the MIC. The time-kill curves revealed that PLS-S1 and -S2 at concentration two-fold above the MIC caused complete killing of the Gram-positive strain *S. aureus* ST1065 within the first 5 min (Fig. 4). By contrast the killing of *S. aureus* ST1065 by PLS-S4 was slower. Almost similar rapid killing kinetics were observed with PLS-S2 and -S4 against the Gram-negative strain *E. coli* ML-35p.

### Permeabilization/disruption of the Gram-positive and Gram-negative bacterial cytoplasmic membranes

**Figure 5:**
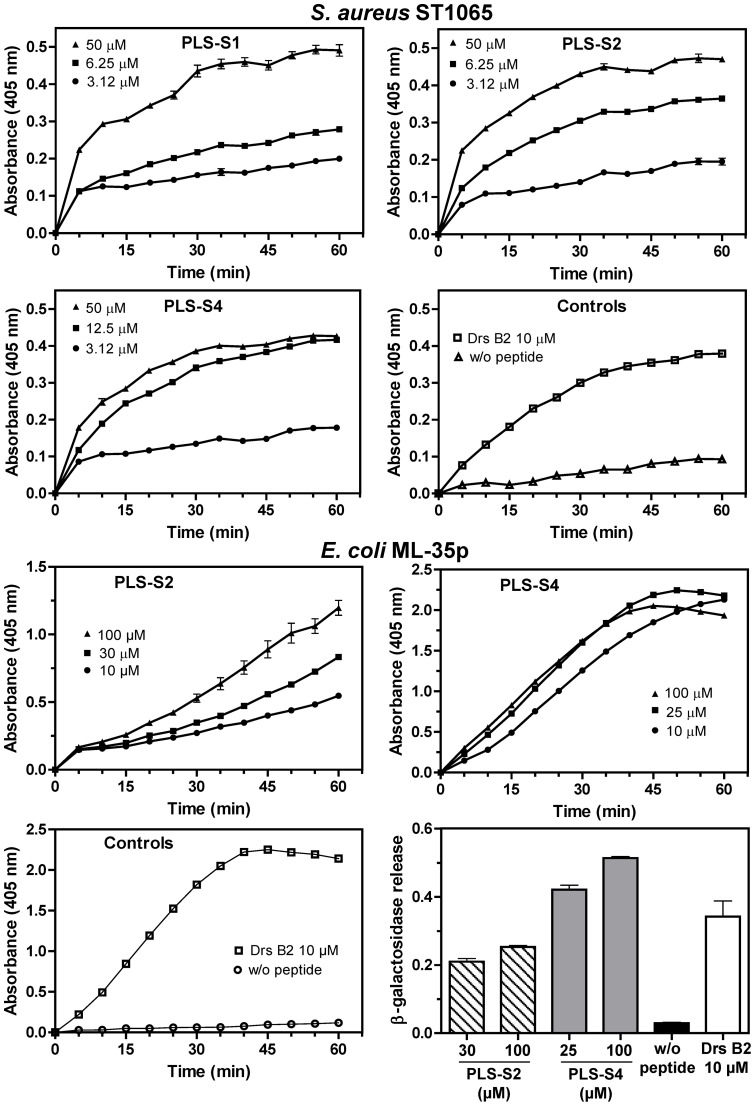
Kinetics of the cytoplasmic membrane leakage of *E. coli* ML-35p and *S. aureus* ST1065 after incubation with different concentrations of active phylloseptins-S. The membrane leakage was determined by measuring the production of o-nitrophenol at 405 nm following hydrolysis of ONPG by the cytoplasmic bacterial β-galactosidase. Data are expressed as the mean ± S.E.M of two experiments carried out in triplicates after subtraction of the negative control values (w/o peptide) from the test values. Control panels display the kinetics obtained without peptide (negative control) and with 10 µM dermaseptin B2 (Drs B2, positive control). For *E. coli* ML-35p, the extracellular release of β-galactosidase was also measured as the production of o-nitrophenol at 405 nm after incubation (60 min) of *E. coli* cells with PLS-S2 (30 and 100 µM) and PLS-S4 (25 and 100 µM), removing of bacteria by centrifugation and adding ONPG to the supernatant. The negative (w/o peptide) and positive (10 µM Drs B2) controls are indicated for comparison. Results are expressed as the mean ± S.E.M of one representative experiment performed in triplicates.

PLS-induced permeabilization of the cytoplasmic membranes of *S. aureus* ST1065 and *E. coli* ML-35p was analyzed by incubating the bacteria, which express β-galactosidase constitutively, with different concentrations of the peptides (*S. aureus* ST1065: PLS-S1, -S2, and -S4; *E. coli* ML-35p: PLS-S2 and -S4) and measuring the time-dependent hydrolysis of the small chromogenic substrate *o*-nitrophenyl-β-D-galactopyranoside (ONPG) into o-nitrophenol (ONP) by cytoplasmic β-galactosidase (Fig. 5). The three peptides permeabilized the bacterial cytoplasmic membrane with increasing severity according to the time and the peptide concentration. The permeabilization was fast and as efficient as with dermaseptin-B2 (10 µM), a 34-residue antimicrobial peptide used as positive control, except for PLS-S2 displaying a lower efficiency against *E. coli* ML-35p. It is notable that the membrane permeabilization occurred even at sublethal peptide concentrations and without any discernible lag, indicating that the peptides can directly permeabilize the cytoplasmic membranes. To investigate the mechanism of permeabilization, *E. coli* ML-35p were incubated with PLS-S2 (30 and 100 µM) and PLS-S4 (25 and 100 µM) for 60 min and β-galactosidase activity was measured after removing bacteria by centrifugation and adding ONPG to the supernatant. As shown in Fig. 5, a dose-dependent release of β-galactosidase was detected, indicating that the membrane became leaky to large cytoplasmic components such as β-galactosidase (∼540 kDa, Stokes radius (Rs)  = 70 Å). A similar behavior was observed for dermaseptin B2 (10 µM) known to act via a carpet-like mechanism [6]. This is in line with the killing kinetics, suggesting that membrane disruption of bacteria by PLS-S1, -S2 and -S4 induces the leakage of the intracellular content and is concomitant with cell death.

### Secondary structure of membrane-bound phylloseptins-S inferred from CD spectroscopy and molecular dynamics

**Figure 6:**
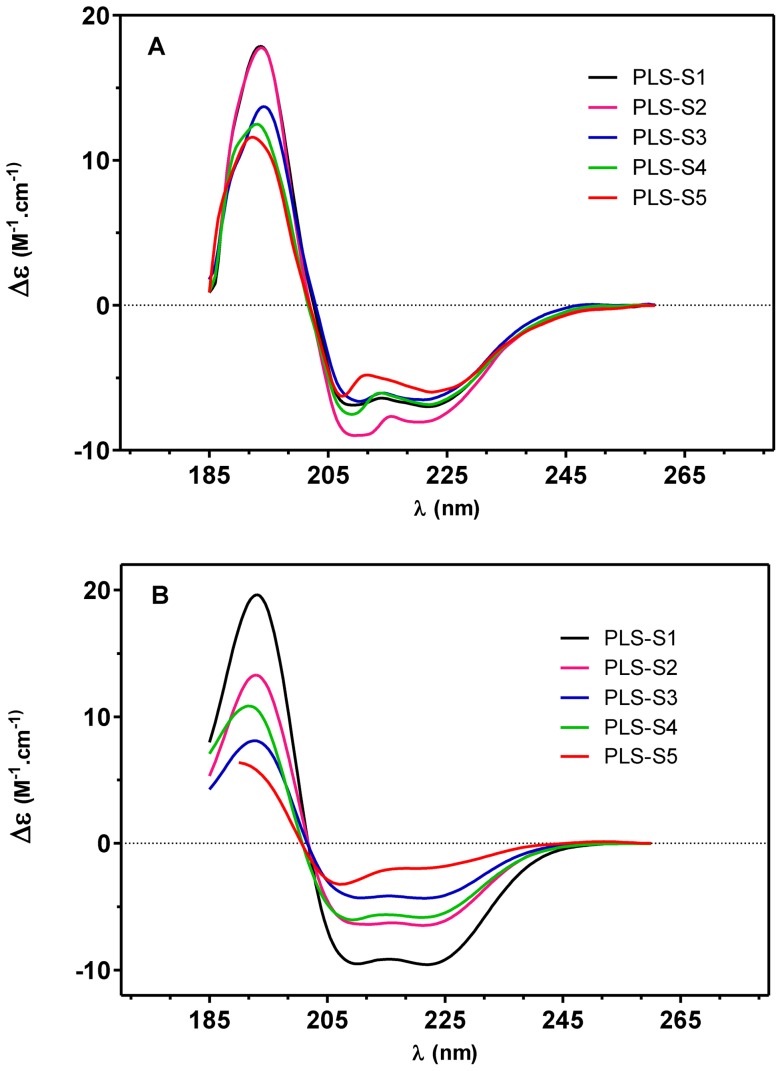
Circular dichroism spectra of synthetic phylloseptins-S (30 µM). (A) DMPC/DMPG (3∶1) large unilamellar vesicles in PBS (1 mg/mL). (B) 80 mM SDS. CD measurements are reported as the dichroic increment (Δε) per residue.

**Figure 7:**
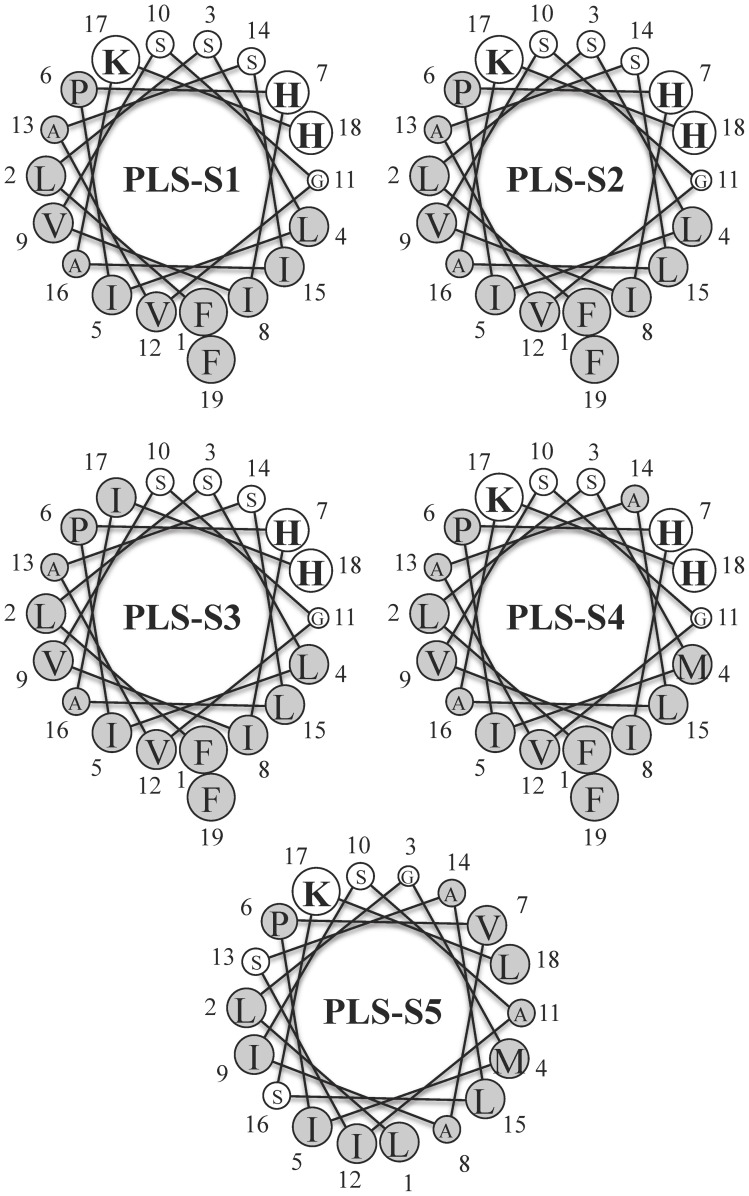
Helical wheel diagram of the different phylloseptins-S. Residues are circled proportionally to amino acid volume. Apolar residues are colored in grey and polar residues are in white. Basic residues are indicated in bold.

**Figure 8:**
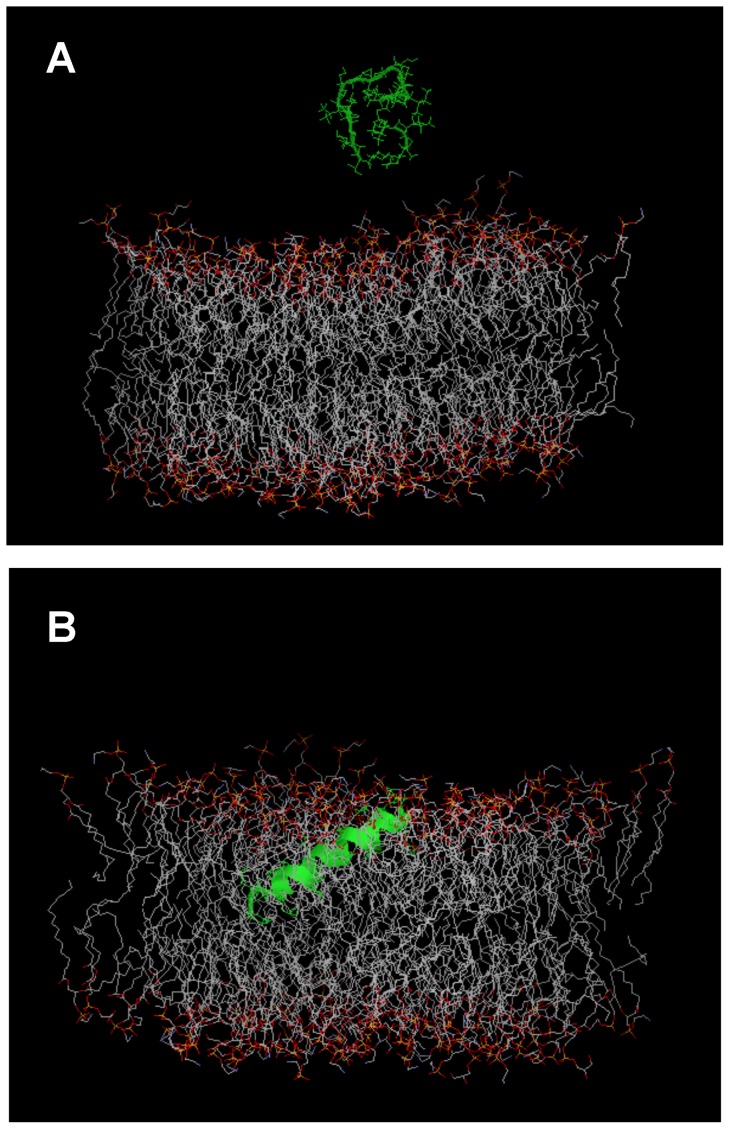
PLS interactions with bacterial membrane studied by molecular dynamic simulations. PLS-S2 was simulated with an *E. coli* membrane bilayer model [26] using the software Hex 6.3. PDB files generated were visualized using Rasmol 2.7.5.2. No docking is observed for the non-structured PLS-S2 (H_2_O) (A) whereas an insertion into the lipid bilayer is predicted for the peptide in α-helical structure (B).

The CD spectra of synthetic PLSs (30 µM) in aqueous solution or in PBS have a single negative band at 198 nm, typical of peptides in random coil conformation (not shown). When mixed with micellar SDS, a membrane-mimetic environment, the spectra are characteristic of an α-helix with a distinct minimum at 222 nm (n−π* transition), a second minimum close to 208 nm (superposition of the random coil π−π* transition at 200 nm and the α-helix π−π* transition at 208 nm), and the α-helix maximum at 193 nm (Fig. 6). The rank order of peptide helix contents was: PLS-S5 (20% helix) < PLS-S3 (44 %) < PLS-S4 (59%) < PLS-S2 (65%) < PLS-S1 (96 %). Helix formation in the presence of DMPC/DMPG (3∶1) large unilamellar vesicles, a bacterial membrane-mimic, follows almost the same trend, i.e. PLS-S5 (60% helix) < PLS-S3 (65% helix) < PLS-S4 (68% helix) < PLS-S1 (70% helix) < PLS-S2 (80% helix). Placing the amino acid sequence of PLSs on a Schiffer-Edmundson wheel projection reveals that the peptides can adopt an amphipathic structure with two separated clusters of hydrophobic and hydrophilic/basic residues located on opposing sides of the helical wheel (Fig. 7). One face of the helix subtending a radial angle of 220–270° perpendicular to the long axis demonstrates a marked hydrophobic character with strongly and bulky apolar residues (Leu, Ile, and Phe) forming a broad and highly prominent hydrophobic patch all along the helix length. The opposite face of the helix containing one to three basic residues (Lys, His) is composed of neutral glycine and small polar residues (Ser). Accordingly, the helical hydrophobic moment of the peptides <µH>, a measure of the amphiphilicity of the helix, was 0.35–0.55, whereas the peptide hydrophobicity <H>  = 0.79–0.95 (Table 1) [29]. We have performed *in silico* studies to visualize the interaction of the potent PLS-S2 with an *E. coli* membrane model. Docking using Hex 6.3 software showed that non-structured PLS-S2 does not interact with the membrane surface whereas α-helical PLS-S2 binds and inserts into the hydrophobic core of the bilayer (Fig. 8).

### Comparing the interactions of phylloseptins-S with zwitterionic and anionic model membranes by differential scanning calorimetry

**Figure 9:**
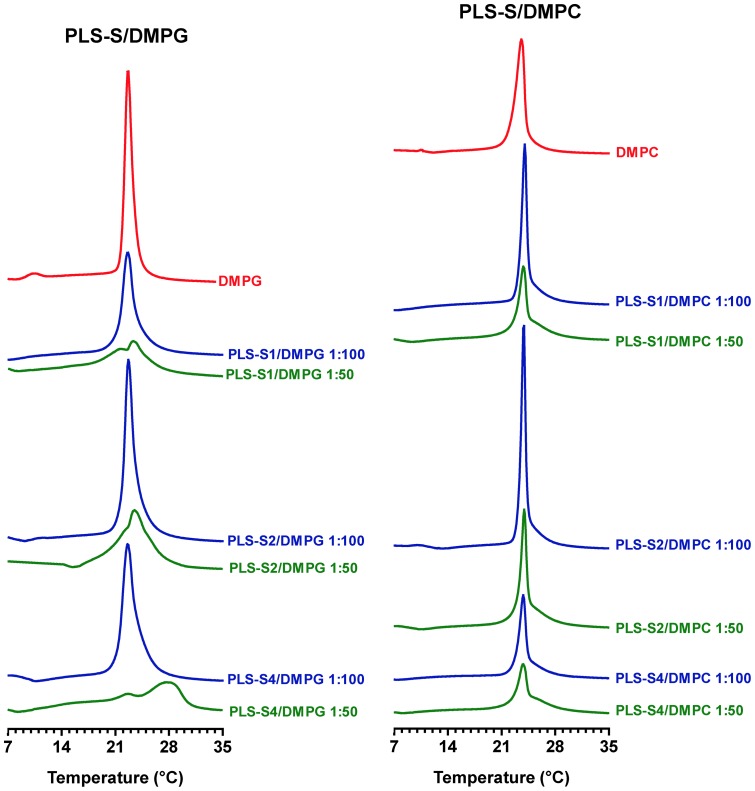
DSC heating thermograms for DMPG and DMPC multilamellar vesicles with or without PLS-S1, -S2 and -S4. Scans were acquired at different peptide/lipid molar ratios (red, lipid control w/o peptide; blue, 1∶100; green, 1∶50;).

Differential scanning calorimetry was used to study the thermotropic behavior of DMPC and DMPG multilamellar vesicles (MLVs) upon addition of PLSs. The peptides were added at different concentrations after liposomes were formed to ensure that they could only interact with the external surface of the MLVs. DMPG was chosen as a model system for bacterial membranes because they contain substantial (up to 30%) amounts of negatively charged lipids such as phosphatidylglycerol and cardiolipin. DMPC, a zwitterionic phospholipid, was used as a model for mammalian cell membranes. In the absence of peptide, DMPC and DMPG MLVs exhibit a strongly endothermic and highly cooperative main transition on heating near 23°C (DMPG) or 24°C (DMPC) (conversion of the rippled gel phase to the fluid lamellar liquid-crystalline phase Lα) [30] (Fig. 9). The main phase transition (chain melting) is mainly due to *trans*-*gauche* isomerization of the acyl chains, which decreases the acyl chain packing of the lipid molecules, increasing fluidity of the membrane. Therefore, the effect of added peptides on the temperature (Tm), enthalpy (ΔH), and cooperativity (ΔT_1/2_) of the main transition serves as an indicator of the ability of the peptide to interact with lipid head groups and to perturb the packing of the lipid acyl chains [31]–[33]. Binding of PLS-S1, -S2 and -S4 (peptide/lipid ratio 1∶100) to negatively charged MLVs strongly reduces the enthalpy of the main phase transition, together with an enhanced broadening of the peak, indicating fluidification of the membrane and lower packing, and van der Waals attraction between lipid molecules as a result of penetration of the peptide into the hydrophobic domain of the bilayer (Fig. 9, Table 4). Increasing quantities of the peptides (peptide/lipid ratio 1∶50) induced a two-component main phase transition, consisting of a broad higher-temperature and less cooperative component superimposed over a sharper lower-temperature component, and a marked decrease (∼50–80%) in the total enthalpy of the main phase transition (the sum of all the components observed). Using the rationale provided by previous studies [34], the sharp low-temperature and broad high-temperature components of the DSC thermograms were assigned to the chain melting phase transition of peptide-poor and peptide-rich phospholipid domains, respectively. The decrease in the temperature and cooperativity of the sharp component may be attributable to domain boundary effects arising from the decreasing size of the peptide-poor lipid domain. These results indicate that PLSs strongly and selectively perturbs anionic bilayer membranes by interacting with the polar head groups and acyl region of the phospholipids, hence disrupting the acyl chain packing of the bilayer. In accordance with their significant hemolytic and cytotoxic activities, binding of PLS-S1, -S2 and -S4 (peptide/lipid ratio 1∶50) to zwitterionic DMPC vesicles also lead to a significant decrease (∼40–70%) in the enthalpy of the main transition (Fig. 9, Table 4). However, no significant effect on the temperature and cooperativity of the main phase transition was observed, indicating that the peptides interacted with the polar head groups and glycerol backbone region of the phospholipids only [6], [25].

**Table 4:** PLS-S effect on the thermotropic behavior of DMPG and DMPC MLVs.

	ΔT_m_,°C	Δ (ΔH),%
**DPMG**		
PLS-S1/DMPG 1:100	0	−53.4
PLS-S1/DMPG 1:50	−1/0.6*	−75.9
PLS-S2/DMPG 1:100	0	−30.2
PLS-S2/DMPG 1:50	−1.1/0.8*	−50.0
PLS-S4/DMPG 1:100	1.1	−25.9
PLS-S4/DMPG 1:50	−0.1/5.1*	−80.2
**DPMC**		
PLS-S1/DMPC 1:100	0.3	−53.3
PLS-S1/DMPC 1:50	0.1	−70.1
PLS-S2/DMPC 1:100	0.2	−19.6
PLS-S2/DMPC 1:50	0.2	−40.8
PLS-S4/DMPC 1:100	0.1	−60.9
PLS-S4/DMPC 1:50	0	−66.9

DMPG: 1,2-dimyristoyl-sn-glycero-3-phosphoglycerol; DMPC: 1,2-dimyristoyl-sn-glycero-3-phosphocholine; MLVs: multilamellar vesicles. Variations of the temperature and total enthalpy values of the main transition phase are indicated as ΔT_m_ (T_m_ – T_m__w/o peptide_) and% Δ(ΔH) [(ΔH – ΔH _w/o peptide_) ×100/ΔH _w/o peptide_]. *ΔT_m_ of the two observed components. T_m_ and ΔH values were estimated by a peak-fitting procedure using CpCalc software and correspond to the mean obtained from six scans.

## Discussion

Phylloseptins-S produced by the skin of *P. sauvagii* are paralogs of similar length (18–19 amino acid residues) but differ markedly in net charge, degree of structure formation, and amphipathicity. They are thus good models for structure-function relationship studies to shed light on the role these parameters play on the ability of these peptides to bind to and disrupt microbial membranes. PLS-S1, -S2 and -S4 have identical net charges (+2), mean hydrophobicities (<H> ∼0.80), and helix-forming propensities (68–80% helix). They adopt an α-helical amphipathic structure in membrane environments (<µH> ∼0.55), with two well-separated clusters of hydrophobic and hydrophilic/basic residues located on opposing sides of the helix. PLS-S1, -S2 and -S4 induced strong perturbations in the chain packing of anionic lipid bilayers that are consistent with deep penetration of the hydrophobic sector of the peptide helix into the fatty acyl chains of the bilayer, with formation of regions of two coexisting phases, one phase rich in peptide and the other lipid-rich. This gradual phase segregation between peptide-poor and peptide-rich domains leads to membrane permeation/disruption. Accordingly, the three peptides show potent bactericidal activities by compromising the structural and functional integrity of the plasma bilayer of the target cells.

Cationic antimicrobial peptides of the α-helical class have a net positive charge of at least +2 [35], which is essential for their mechanism of action because the positively charged polar face of the peptide helix will drive the initial attraction to the negatively charged components of the microbial membrane through long range electrostatic interactions. PLS-S2 and PLS-S3 only differ by one amino acid. Whereas PLS-2 carries a lysine in position 17, an isoleucine is located at the same position of PLS-3. Although the two peptides have similar overall helix-forming propensities (65–80% helix) and amphipathicities (<µH>  = 0.43–0.55), the most pronounced difference is the variation of their net charge at physiological pH. Whereas PLS-S2 (net charge  = +2) was active against most of the tested microorganisms at µM concentrations, PLS-S3 (net charge  = +1) was virtually inactive. This adds further support to the view that a positive net charge of at least +2 is a key factor for potent antimicrobial activity in α-helical amphipathic peptides. Although histidine side chain has a pKa value close to 6.5 when in an unstructured polypeptide chain, thus being only weakly charged at pH ≥7, note, however, that the ionization state of the His residue can be modulated by hydrophobic, electrostatic and polar interactions with anionic surfaces. For instance, a large shift of pKa of the His residue to a much higher value is expected if His acts as a hydrogen bond donor to form a H-bond with the anionic head groups of negatively charged bilayers via its Nδ1H^+^ or Nε2H^+^ groups [36].

A fine balance of electrostatic and hydrophobic interactions is needed to assure for membrane association and formation of an amphipathic helical structure with a high hydrophobic moment, thereby promoting membrane insertion and disruption. There is good agreement that cationic α-helical AMPs are attracted by the bacterial membranes by electrostatic interactions and then undergo conformational changes during membrane interactions. The coil-helix transition contributes about 0.5 kcal/mol per residue to the membrane binding energy of amphipathic peptides [37], which should promote the membrane association. Once bound, with its helix axis parallel to the bilayer surface, the peptide would insert the hydrophobic face of the helix within the membrane interior through hydrophobic and van der Waals interactions, causing local fusion of the membrane leaflets, pore formation, cracks and membrane disruption.

PLS-S5 has a net charge of +2 but is virtually inactive against microorganisms. Among the phylloseptins-S, PLS-S5 has the lowest helix-forming propensity and amphipathicity. It is thus likely that the differences in antimicrobial efficacies of PLS-S5 and PLS-S1, S2 and -S4 may reflect differences between the degree of helix formation and/or amphipathicity of these peptides. First, two basic residues (Lys^17^ and His^18^) are found for PLS-S1, -S2 and -S4 at the C-terminus of the helix. As shown by Resende et al. [17] and previous investigations [38], the interactions of the helix dipole with a positively charged side chain, histidine in particular, positioned at the C-terminus of the helix have strong helix stabilizing properties. This effect is less pronounced for PLS-S5 that carries only one lysine residue in this region. An additional energy contribution to helix stabilization of PLS-S1, -S2 and -S4 versus PLS-S5 invokes cation-π interactions between His^18^ and Phe^19^ that could promote helical conformations at the C-terminus [17], [39].

Second, PLS-S1, -S2 and -S4 form well-behaved amphipathic α-helices once bound to anionic membrane-mimetics. One face of the helix demonstrates a marked hydrophobic character with strongly and bulky apolar residues (Leu, Ile, and Phe) forming a highly prominent and uninterrupted hydrophobic patch all along the helix length. Phe residues are known to act as amphipathic in-plane membrane anchors [40]. The opposite face of the helix is composed by an alternation of basic residues, neutral glycine and polar residues (Ser). Accordingly, the helical hydrophobic moment of the peptide <μH>, a measure of the amphiphilicity of the helix, is 0.52–0.55. In contrast, the polar helical surface of PLS-S5 is rather small, being constituted of only a lysine, a glycine and a serine. In addition, two polar residues (Ser) interrupt the continuity of the opposite shallow apolar face of the helix cylinder. Accordingly, the helix formed by PLS-S5 has very low amphipathicity (<µH> = 0.35).

Phylloseptins-S displayed antibacterial activities against Gram-negative and Gram-positive bacteria, as well as antiparasitic activity against promastigote of *Leishmania* sp. They however exhibit also a significant toxicity against mammalian cells. Hydrophobicity of cationic α-helical amphipathic peptides appears to have a higher impact on mammalian cell toxicity than on antibacterial action. Highly hydrophobic peptides are related to higher hemolysis and a decrease in discrimination between host cytotoxicity and antimicrobial activity. Phylloseptins-S are highly hydrophobic and only mildly cationic. Although basic peptides interact much better with anionic membranes, and to a lesser degree with zwitterionic membranes, it has been shown that hydrophobic effects should account for the binding capacity of neutral peptides to anionic lipids [36]. This stresses the importance of hydrophobic interactions between the peptides and the lipid bilayer for helix formation and stabilization.

## Supporting Information

Figure S1 **MALDI-TOF MS spectrum of the HPLC fractions containing (A) PLS-S4 (fraction 53 min), (B) PLS-S1/S2 (fraction 54 min), and (C) PLS-S5 (fraction 62 min).** The [M+H]^+^ ion of the corresponding mature peptides is indicated with a circle. Lower panel, zoom of the MS spectrum showing sodium and potassium adducts (PLS-S1/S2 and PLS-S5) and also the oxidation of the methionine (PLS-S5, Ox Met: methionine sulfoxide).
(TIFF)

Figure S2 **MALDI-TOF-TOF MS/MS spectra obtained from collision-induced dissociation of the phylloseptins-S parent ions.** (A) PLS-S4, [M+H]^+^ = 2003; (B) PLS-S1/S2, [M+H]^+^ = 2035; (C) PLS-S5: [M+H]^+^ = 1796. Lower panel, b- and y-ion series. For each PLS-S, the sequence was obtained with remaining leucine/isoleucine indeterminations.
(TIFF)
