# Correction: Structure, Antimicrobial Activities and Mode of Interaction with Membranes of Bovel Phylloseptins from the Painted-Belly Leaf Frog, *Phyllomedusa sauvagii*

**DOI:** 10.1371/annotation/dbb3e614-dc4c-40dd-b9e0-37787ae6b150

**Published:** 2013-08-27

**Authors:** Zahid Raja, Sonia André, Christophe Piesse, Denis Sereno, Pierre Nicolas, Thierry Foulon, Bruno Oury, Ali Ladram

There was an error in the article title.

The correct title is: Structure, Antimicrobial Activities and Mode of Interaction with Membranes of Novel Phylloseptins from the Painted-Belly Leaf Frog, *Phyllomedusa sauvagii*

The correct citation is: Raja Z, André S, Piesse C, Sereno D, Nicolas P, et al. (2013) Structure, Antimicrobial Activities and Mode of Interaction with Membranes of Novel Phylloseptins from the Painted-Belly Leaf Frog, *Phyllomedusa sauvagii*. PLoS ONE 8(8): e70782. doi:10.1371/journal.pone.0070782 

